# Case Report: High burdens of air sac worms (*Diplotriaena* sp.) in three northern flickers (*Colaptes auratus)* and a pileated woodpecker (*Dryocopus pileatus*)

**DOI:** 10.3389/fpara.2025.1547153

**Published:** 2025-03-21

**Authors:** Alyssa R. Freeman, Lyndon E. Sullivan-Brugger, Bethany Groves, Nicki Rosenhagen, Kayla B. Garrett, Michael J. Yabsley

**Affiliations:** ^1^ Warnell School of Forestry and Natural Resources, University of Georgia (UGA), Athens, GA, United States; ^2^ Southeastern Cooperative Wildlife Disease Study Laboratory, The University of Georgia, Athens, GA, United States; ^3^ Progressive Animal Welfare Society (PAWS) Wildlife Rehabilitation Center, Lynnwood, WA, United States; ^4^ Center for Emerging Infectious Diseases, Athens, GA, United States

**Keywords:** Diplotriaenidae, nematode, parasite, wildlife rehabilitation, woodpecker

## Abstract

*Diplotriaena* spp. are nematode parasites of the abdominal and thoracic air sacs of numerous avian species worldwide. *Dipoltriaena* infections are generally subclinical, but high worm burdens can lead to morbidity and mortality. In this case series, *Diplotriaena* were recovered from a pileated woodpecker (*Dryocopus pileatus*) in 2017 and three northern flickers (*Colaptes auratus*) in 2023 and 2024 from Washington, USA. All four presented to a wildlife rehabilitation center with either respiratory signs or trauma with varied severity. A large number of worms (>44 worms) were surgically removed from the pileated woodpecker. The bird improved and was subsequently released. All three northern flickers were humanely euthanized due to poor prognosis and worsening conditions. Nematodes from Cases 1 and 4 were identified as a *Diplotriaena* sp. but they did not match any described species. Ethanol-fixed worms were available from one flicker case for genetic characterization. Partial 18S rRNA sequences (888bp) from two worms from a flicker were identical and 98-98.5% similar to numerous *Diplotriaena obtusa* sequences. The sample *Diplotriaena* sp. grouped separately from the three closest matches in the GenBank database, *Diplotriaena anthreptis* and two clades of *Diplotriaena obtusa* and *Diplotriaena bargusinica.* The partial COI sequences (674bp) were identical to each other and ~80-85% similar to numerous Spiruromorpha representatives. Due to a lack of available samples in the GenBank database and incomplete morphological descriptions of the genus, identification to species was not possible. In summary, all four cases in this case series occurred in free-ranging birds in Washington state and represented unusually high burdens of *Diplotriaena* sp. We believe that the high worm burden contributed to trauma, respiratory pathology, and weight loss. Additional surveillance is needed to determine the prevalence and impact of this parasite on woodpecker populations and to more accurately identify the parasite species in these two species of woodpeckers.

## 1 Introduction

Northern flickers (*Colaptes auratus*) and pileated woodpeckers (*Dryocopus pileatus*) (Order: Piciformes) are native North American woodpeckers. They are parasitized by air sac parasites of the genus *Diplotriaena* (Nematoda: Diplotriaenidae, Order Spirurida) which are transmitted by consumption of infected intermediate host arthropods such as orthopterans. Historically, *Diplotriaena* spp. were thought to be specific to corvids (Cawthorn and Anderson, 1980a); however, they have since been reported globally in multiple avian orders including Anseriformes, Apodiformes, Columbiformes, Piciformes, and Passeriformes (Barus, 1971; Mobedi et al., 2006; Sterner and Cole, 2008; Borji and Razmyar, 2011; Chandio et al., 2015; Hong et al., 2019; Michalski et al., 2021; Rentería-Solís et al., 2021). Currently, there are approximately 77 described species of *Diplotriaena* (Hong et al., 2019), of which, two have been reported in Piciformes: *Diplotriaena serratospicula* in a Cuban green woodpecker (*Xiphidiopicus percussus*) (Barus, 1971) and Hispaniolan woodpecker (*Melanerpes striatus*) (Wehr, 1934) and *Diplotriaena americana* in a West Indian woodpecker (*Melanerpes superciliaris*) (Barus, 1971) and a northern flicker (Walton, 1927).

The life cycle of *Diplotriaena* spp. begins when embryonated eggs are passed in the feces of infected birds and consumed by an Orthopteran intermediate host (grasshoppers) (Sterner and Cole, 2008). The larvae hatch and burrow into the main body cavity, where they develop to infective third-stage larvae (L3). Once an L3-infected grasshopper is ingested, *Diplotriaena* larvae emerge and burrow out of the bird’s intestines, molting into fourth-stage larvae (subadults) when they enter the hepatic portal system. They migrate to the air sac through the heart, pulmonary arteries, and lungs where they undergo their final molt into adults and reproduce. Adults parasitize the air sacs, lungs, and body cavity. Parasite eggs are coughed up by the host, swallowed, and subsequently passed in the feces.

*Diplotriaena* infections are generally subclinical, but high worm burdens can cause non-specific signs such as lethargy, dyspnea, trembling, poor body condition, unthriftiness, and reduced flight performance (Sterner and Cole, 2008). These clinical signs can be associated with larval migration in early infections or high burdens of adult worms in the air sacs in chronic infections. In some cases, *Diplotriaena* infections can result in chronic secondary bacterial or fungal infections (Sterner and Cole, 2008; Young et al., 1963). Here, we report four severe cases of *Diplotriaena* infections in two free-ranging picid species (one pileated woodpecker and three northern flickers) from Washington state, USA.

## 2 Case descriptions

### 2.1 Case 1

**Figure 1:**
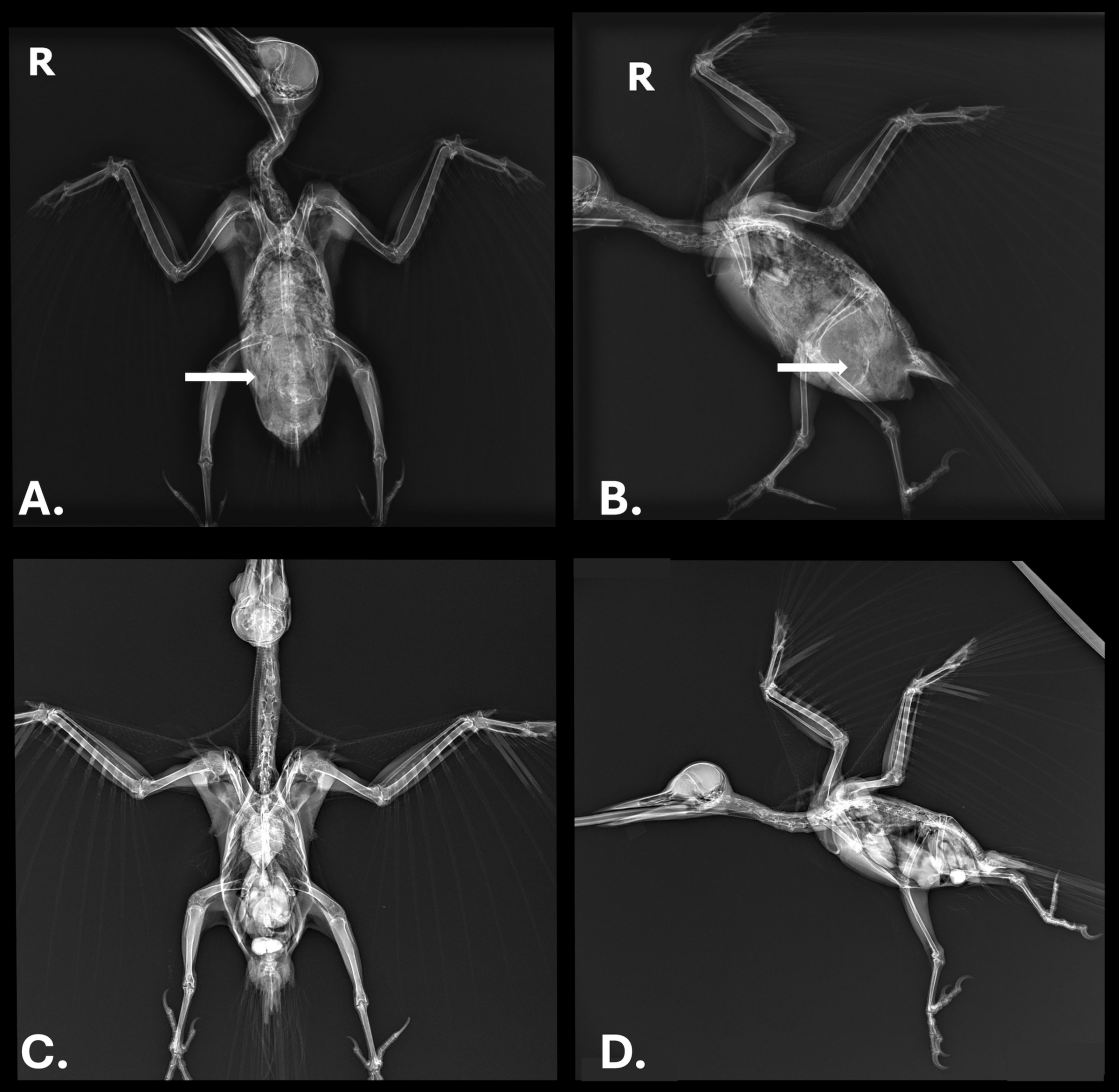
**(A)** Ventrodorsal view and **(B)** lateral view radiographs of a pileated woodpecker (*Dryocopus pileatus*) with high burden of a *Diplotriaena* sp. White arrows represent where air sac worms are present; only one side in A is marked for simplicity. **(C, D)** Radiographs of an uninfected pileated woodpecker showing the same region where air sacs are clear instead of opaque.

**Figure 2:**
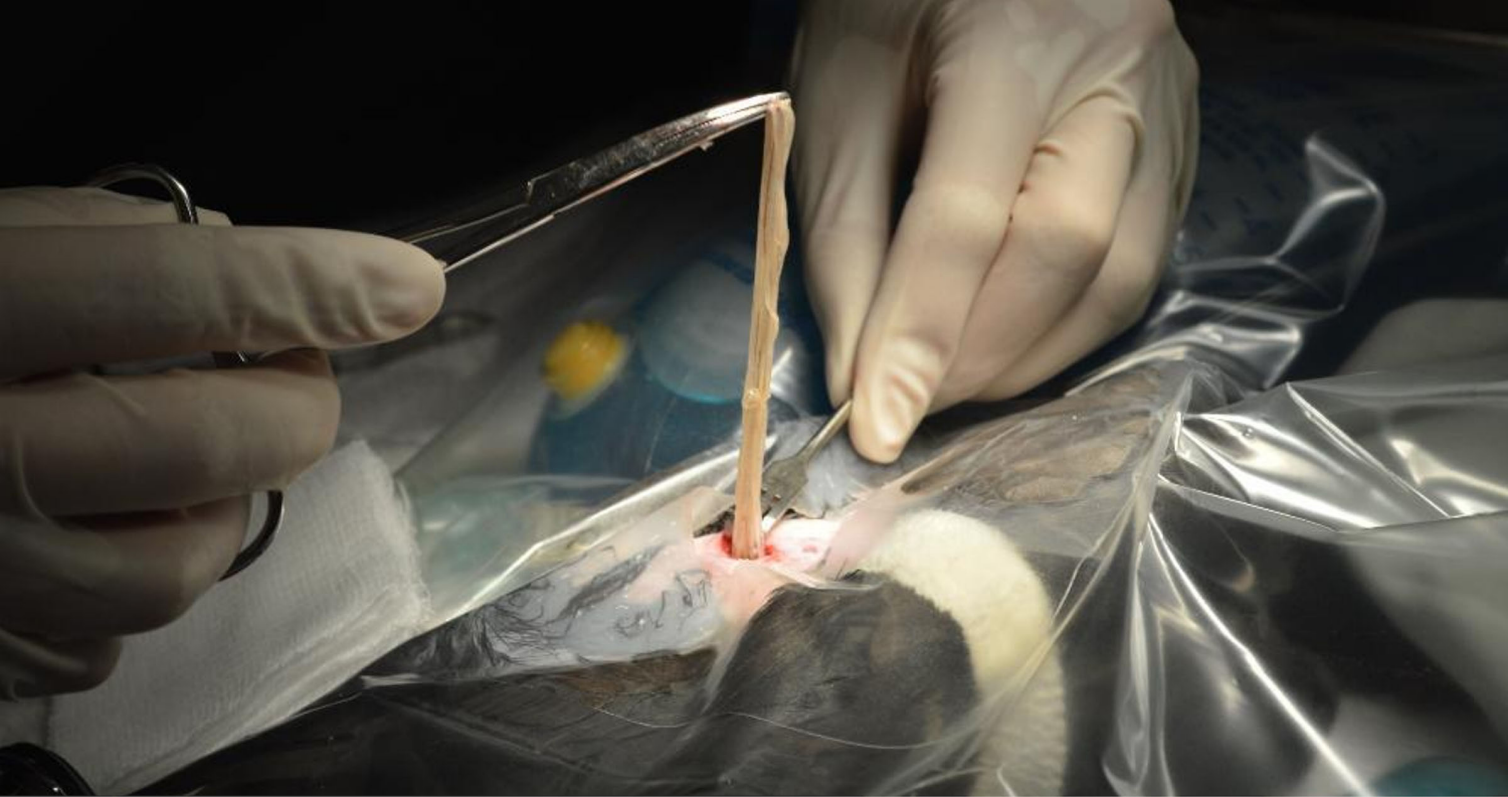
Surgical removal of numerous *Diplotriaena* sp. worms from the abdominal air sac of a pileated woodpecker (*Dryocopus pileatus*).

In August 2017, a 322 g free-ranging adult male pileated woodpecker was found demonstrating labored respirations and in unthrifty condition in King County in western Washington state in the northwestern United States. Upon examination, the woodpecker also displayed wheezing, had visible bruising over the abdomen, and white worms were observed moving subcutaneously in the ventral coelomic region. A fecal flotation with zinc sulfate solution on the day after admission revealed a massive number of nematode eggs (capillarid eggs, ascarid-type eggs, and eggs morphologically consistent with *Diplotriaena* [see Section 3.1 for measurements]). Repeat fecal flotations on days 3 and 16 after admission were still positive for capillarid and *Diplotriaena* eggs). Treatment consisted of amoxicillin and clavulanate (0.32ml, 62.5 mg/ml) bis in die (BID; twice per day) per os (PO; by mouth) for four days, ivermectin (0.12 ml, 1 mg/ml) PO on days 1 and 10 after admission, and subcutaneous fluids (15 ml, 2.5% dextrose/Lactated Ringer’s Solution) BID for three days. While in care, the woodpecker exhibited open-beak breathing and dyspnea yet remained active. After several days with no change in condition and weight loss (down to 298 g), radiographs were taken and revealed significantly increased soft tissue opacity in the lungs and air sacs, especially in the caudal half of the body (**Figure 1**). The lungs had an almost bronchiolar appearance (i.e., thickened rings with air lucency in the center) while the air sacs were more diffusely opacified (**Figure 1**). Surgical removal of whole and fragmented nematodes was conducted from the abdominal air sac under general anesthesia (**Figure 2**); however, many worms were observed and left in the bird due to inaccessibility. Details on the morphology of the worms is provided in Section 3.1 below. After surgery, the bird showed continued improvement and weight gain (316 g) until seven days after surgery when the patient was released at the point-of-origin.

### 2.2 Case 2

In April 2023, a free-ranging adult male northern flicker from King County, Washington was admitted after being attacked by a domestic house cat. An initial physical examination found the patient alert and in good body condition. Several small soft tissue wounds and bruising were present on the body and wings. Radiography revealed reduced space in the air sac and a distended coelom. A fecal flotation with zinc sulfate solution was positive for eggs morphologically consistent with *Diplotriaena*, but the bird had no signs of respiratory distress. The patient was treated with amoxicillin and clavulanate (0.32ml, 62.5 mg/ml) BID PO for four days, ivermectin (0.01 ml, 10 mg/ml) PO for two days, praziquantel (0.03 ml, 56.8 mg/ml) PO for two days, and toltrazuril (0.03 ml, 50mg/ml) PO for three days. The patient was also given meloxicam (0.11 ml, 1.5mg/ml) BID PO for three days after admission and gabapentin (0.04 ml, 100mg/ml) BID PO was given for four days starting on day 2 after admission. By nine days post-admission, the flicker lost 25% of its body weight (162 g to 122 g), displayed a decline in body condition score, and developed neurologic signs. With no improvement, the flicker was humanely euthanized with an overdose (0.20 ml) of intravenous (IV) pentobarbital sodium (Fatal Plus, Vortech Pharmaceuticals, Ltd.). At necropsy, large numbers of nematodes were detected in the air sac, but none were collected.

### 2.3 Case 3

In April 2023, a free-ranging adult male northern flicker in good body condition from Snohomish County (located north of King County) in western Washington was admitted for a suspected hit-by-vehicle with wet respirations and an inability to fly. Radiography revealed a fractured keel and sternum. No fecal exam was completed. The patient was treated with ivermectin (0.06ml, 1mg/ml) PO for nine days, praziquantel (0.02 ml, 56.8mg/ml) PO for three days, and toltrazuril (0.03 ml, 50mg/ml) PO for four days. The patient was also given meloxicam (0.1ml, 1.5mg/ml) BID PO and tramadol (0.02 ml, 25 mg/ml) BID PO from admission to euthanasia. During the first four days in care, the body condition score declined from good to poor, and the bird required tubing after a 20% loss in body weight (156 g to 123.8 g). Due to abnormally delayed healing time for the identified orthopedic injuries, ongoing exercise-induced respiratory distress, and development of subcutaneous emphysema, the bird was humanely euthanized with 0.2ml IV pentobarbital sodium (Fatal Plus). At necropsy, multiple adjacent displaced rib fractures along the left side of the coelom and diffuse emphysema along the body wall were observed. Cranial intracoelomic adhesions between the ribs, lungs, and heart were evident with hemorrhage in the oral cavity, trachea, and lungs. A high worm burden (exact number not recorded) was present in the caudal air sacs.

### 2.4 Case 4

**Figure 3:**
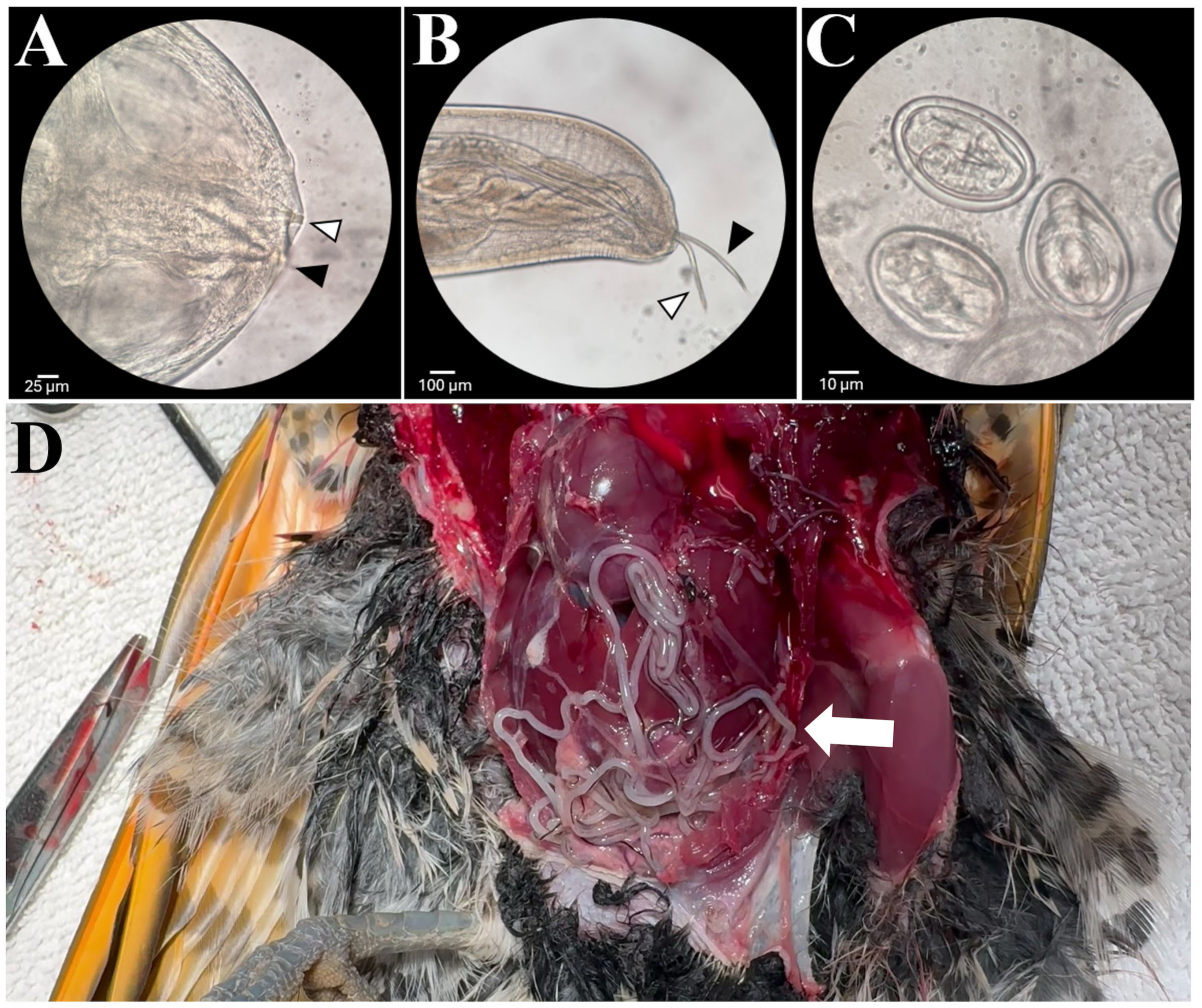
**(A)** Anterior end of a female worm showing the three-pronged tridents. **(B)** Caudal end of a male worm removed from the northern flicker showing the long (black arrow) and short (white arrow) spicules. **(C)** *Diplotriaena* eggs removed from an adult worm. **(D)** Numerous *Diplotriaena* worms in the air sacs of a northern flicker *(Colaptes auratus)* at necropsy.

In February 2024, a 138 g free-ranging adult male northern flicker from King County, Washington was admitted for inability to fly with evidence of a left shoulder injury and moderate-to-severe diffuse subcutaneous emphysema. Radiography confirmed a fracture of the left coracoid with mild to moderate caudal displacement of the left shoulder, scattered subcutaneous emphysema, and severe loss of air sac space in the coelom with abnormal opacities present. A routine fecal flotation with zinc sulfate solution was performed and was positive for nematode eggs morphologically consistent with *Diplotriaena*. The patient was treated with ivermectin (0.06ml, 1mg/ml) PO for one day, praziquantel (0.02 ml, 56.8mg/ml) PO for three days, and toltrazuril (0.03 ml, 50mg/ml) PO for three days. The patient was also given meloxicam (0.09ml, 1.5mg/ml) BID PO and tramadol (0.02 ml, 25 mg/ml) BID PO from admission to euthanasia The patient increased in weight (148 g) following increased nutritional support but failed to regain flight ability due to the severity of the shoulder injury. The bird was humanely euthanized using 0.2 ml IV pentobarbital sodium (Fatal Plus). At postmortem examination, 51 nematodes were removed from the thoracic and abdominal air sacs (**Figure 3**).

## 3 Diagnostics assessment

### 3.1 Morphological identification

A total of 44 worms (35 whole worms and nine fragments; all female) were collected during surgery of Case 1 (although some were left in the bird) and 51 (46 females and 5 males) were collected from Case 4 at necropsy. Numerous worms were observed at necropsy of Cases 2 and 3 but were not collected so a count was not possible. Nematodes collected from Cases 1 and 4 were identified as a *Diplotriaena* sp. based on several morphological features including size, anterior and posterior features, and presence and morphology of the large paired chitinous tridents (curvature of the trident’s apex), a taxonomically important structure in the genus *Diplotriaena* (**Figure 3**) (Hong et al., 2019; Wong et al., 1983).

Generally, the morphology of female nematodes collected from Cases 1 (pileated woodpecker) and 4 (northern flicker) were similar to each other but did vary in the trident length and neither matched any described species included in the keys by Seibert (1944) and Anderson (1959). Female worms from the pileated woodpecker and northern flicker measured from 118-198 mm and 140-210 mm, respectively, in total body length. Males from Case 4 were significantly smaller, measuring 43-52 mm long (**Supplementary Table S1**).

The worms were cleared with an 80% phenol:20% ethanol solution, mounted on glass slides, and observed with a Nikon ALPHAPHOT-2 YS2 microscope to observe internal structures and obtain measurements. Measurements for trident length, distance from anterior end to the nerve ring, total esophagus length as well as lengths of anterior and posterior portions of the esophagus are provided in **Supplementary Table S1**. In addition, distance from the anterior end to the vulva for worms from both woodpecker species and the length of the unserrated spicules for males from a northen flicker are reported in **Supplementary Table S1**. Eggs (n=5) from five different female worms from the pileated woodpecker and northern flicker measured an average of 48 μm x 35 μm (length:width ratio 1.38) and 48 μm x 37.5 μm (L:W ratio 1.28) (**Supplementary Table S1**).

### 3.2 Molecular analysis

Molecular analysis was only possible on worms from one of the flickers (Case 4) because parasites from other cases were either not collected (Cases 2 and 3) or were preserved in 10% neutral buffered formalin (Case 1). Genomic DNA was extracted from two nematodes using a QIAGEN DNeasy Blood and Tissue Extraction Kit (Germantown, MD, USA) following the manufacturer’s directions. Portions of the 18S rRNA and cytochrome *c* oxidase I (*COI*) genes were amplified using primers 18S39F and 18S977R (Olson et al., 2017) and a cocktail of three primer sets, respectively (Prosser et al., 2013). Amplicons were detected in a 1% agarose gel stained with GelRed (Biotium, Fremont, CA, USA) and extracted from the gel using a QIAGEN gel extraction kit. Amplicons were Bi-directionally sequenced at Genewiz (South Plainfield, NJ, USA). Sequences were edited and assembled, primer sequences removed, aligned with related sequences from GenBank, and a phylogenetic tree was constructed using an approximately maximum-likelihood method with FastTree v2.1 with a generalized time-reversible (GTR) model in Geneious Prime 2024.0.7 (Biomatters Limited, Auckland, New Zealand). Some 18S rRNA sequences were shorter than our available sequence so two analyses were conducted, one with the full 888bp of our sequence and another with additional sequences in GenBank, but only included 503bp. Members of the Desmidocercidae were selected as an outgroup based on previous work (Vanstreels et al., 2018; Bennett et al., 2022).

**Figure 4:**
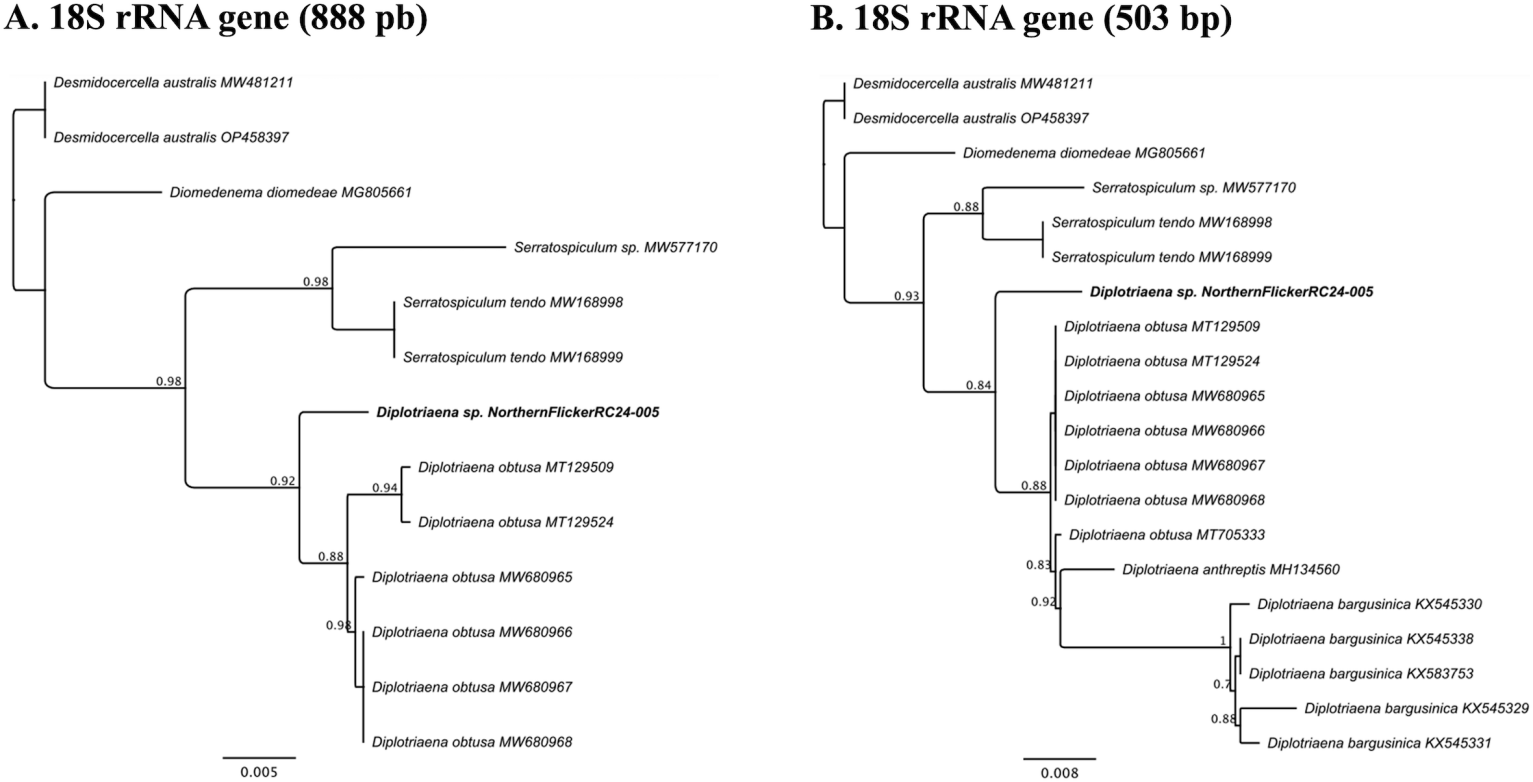
**(A)** Phylogenetic tree produced by FastTree v2.1 for the 18S rRNA gene (888bp) of a *Diplotriaena* sp. from a northern flicker (*Colaptes auratus*) and related species. **(B)** Phylogenetic tree produced by FastTree v2.1 for a shorter region of the 18S rRNA gene (503bp) of a *Diplotriaena* sp. from a northern flicker and related species. For both figures, the sample from the northern flicker is identified in bold. Also, for both analyses, *Desmidocercella australis* was used as an outgroup.

The partial 18S rRNA sequences (888bp) from the two worms from the flicker were identical and were 98-98.5% similar to numerous *Diplotriaena obtusa* sequences in GenBank. Comparison of our sequence with the shorter (503bp) sequences available for other *Diplotriaena* spp. in GenBank showed that the flicker sequence was most similar to *D. obtusa* (98-98.5%), followed by *Diplotriaena anthreptis* (98.1%) and *Diplotriaena bargusinica* (96.2-96.8%). Phylogenetic analyses showed that the flicker *Diplotriaena* sp. grouped separately from *D. anthreptis* and two clades of *D. obtusa* and *D. bargusinica* (**Figure 4**). The partial COI sequences (674bp) were identical to each other and ~80-85% similar to numerous Spiruromorpha representatives. Phylogenetic analyses were limited because few closely related species have sequences in GenBank, but it grouped with *Serratospiculum tendo*, similar to the 18S rRNA analysis (**Supplementary Figure S1**). Unique sequences were submitted to GenBank (accession numbers PV179416-PV179417).

## 4 Discussion

We present data on four cases of severe *Diplotriaena* infections in two woodpecker species in Washington state, USA. All four presented to a wildlife rehabilitation center with either respiratory signs or trauma with varied severity. Infections with *Diplotriaena* and related air sac nematodes are often reported in birds with no associated clinical signs, but large burdens can lead to morbidity and mortality with traumatic injuries often being reported (Cawthorn and Anderson, 1980b; Hawkins et al., 2001; Okulewicz and Sitko, 2012; Santoro et al., 2016; Van Wettere et al., 2018; Michalski et al., 2021). Mild to severe respiratory signs were observed in the pileated woodpecker and two of the three northern flickers documented here, which can predispose birds to traumatic events (e.g., being unable to avoid car strikes or domestic cat attacks). One northern flicker (Case 4) showed no obvious signs of respiratory distress despite the post-mortem surgical removal of over 50 worms from the caudal and thoracic air sacs. However, this bird did have periods when it was fluffed and had its head tucked that could have been due to pain or distress. Three of the four cases demonstrated weight loss and reduced fat reserves. The three flickers were all euthanized due to poor prognosis or declining health, but all three flickers also had mild to severe traumatic lesions. It is unknown if the heavy worm burdens contributed to trauma, but lethargy, depressed respiratory ability, and poor flight performance due to the parasitic infection seem likely to be a contributing factor (Sterner and Cole, 2008). This is consistent with observations of *D. obtusa-*infected cliff swallows (*Petrochelidon pyrrhonota*) having high reports of blunt force trauma likely associated with lowered function (Michalski et al., 2021).

*Diplotriaena* infections in birds are often incidentally detected at necropsy. In general, diagnosis of parasitic infections in live birds relies on the detection of eggs in feces or visual observation of worms (Seibert, 1944; Wyckoff et al., 2023). However, detecting eggs in feces can be difficult due to low numbers of eggs shed, inconsistent shedding, or inability to identify eggs to species (Ballweber et al., 2014; Greiner, 1989). In three of the four cases, a fecal exam was conducted, and nematode eggs were readily detected, possibly due to the large worm burdens present. In addition, radiographs were obtained from three of the four cases, and all three showed loss of air space and other lesions due to the parasites. The pileated woodpecker survived following the surgical removal of numerous worms; however, only one pileated woodpecker was admitted, so we do not know if this parasite is more pathogenic to northern flickers or if they presented with more severe pathology that resulted in their poor prognosis. Additionally, unlike the northern flickers, the pileated woodpecker was admitted without obvious traumatic injuries. Regardless, early detection and subsequent surgical intervention to address high worm burdens may increase the survival of severely infected birds. Data on treatment efficacy for air sac parasites are limited, and although falcons infected with related worms (*Serratospiculum*) have been treated with ivermectin and fenbendazole, data for *Diplotriaena* are lacking (Al-Timimi et al., 2009; Veiga et al., 2017). However, even after treatment, worms may need to be surgically removed to minimize inflammation associated with dead parasites (Samour and Naldo, 2001).

All four cases had worms that were grossly morphologically consistent with *Diplotriaena* but worms were only available from two cases for microscopic evaluation. Identification of different *Diplotriaena* spp. is often difficult due to a lack of complete descriptions for some species, difficulty in detecting some identifying characteristics, and lack of male worms. Common morphological traits used to distinguish *Diplotriaena* species include body size, texture of body, number of caudal papillae, size and shape of spicules, trident morphology, and size of eggs (Borji and Razmyar, 2011; Hong et al., 2019; Michalski et al., 2021). The worms from Case 1 (pileated woodpecker) were preserved in 10% neutral buffered formalin since 2017 so efforts to extract DNA failed. Also, this case had only female worms present. The only differences in standard morphological measurements between the female worms from Cases 1 and 4 was trident length and neither matched measurements for the only other two *Diplotriaena* species (*D. americana* and *D. serratospicula*) reported from woodpeckers (**Supplementary Table S1**). In addition, the short spicule length from our northern flicker case was significantly shorter than the spicule length of *D. americana* described from a northern flicker in Cuba (Walton, 1927). Future molecular work combined with morphological data would be useful to determine if the same parasite species infects both species of woodpeckers and how they are related to *D. americana* and *D. serratospicula*. The molecular analysis of worms from Case 4 confirmed the worms were *Diplotriaena* but did not match any species in GenBank; however, there is a paucity of species (n=3) represented in GenBank and sequences of *D. bargusinica* are all short (500bp). This is especially poor given there are at least 77 described *Diplotriaena* spp. Ideally, new descriptions should include both morphologic and molecular data. There is also a need to obtain new molecular data for parasites that have limited species descriptions or when there may be species complexes. Because the genus *Diplotriaena* is so speciose, any new molecular data for this group is important. In the future, sequences from more representatives of this understudied group of parasites are needed to understand their host-relationships.

In summary, all four cases in this case series occurred in free-ranging birds within a small region (King and Snohomish counties) of Washington state and represented unusually high burdens of *Diplotriaena* sp. Although presentation of clinical signs varied between individuals, it is suspected that the high worm burden contributed to trauma, respiratory pathology, and weight loss. It is unknown if this area has an unusually high prevalence for this parasite because there has not been a systematic survey for *Diplotriaena* in woodpeckers in the region. Additional surveillance is needed to determine the prevalence and impact of this parasite on woodpecker populations and to more accurately identify the parasite species in these two species of woodpeckers.

## Data Availability

The datasets presented in this study can be found in online repositories. The names of the repository/repositories and accession number(s) can be found in the article/**Supplementary Material**.
