# Supplementary material for: Case Report: High burdens of air sac worms (Diplotriaena sp.) in three northern flickers (Colaptes auratus) and a pileated woodpecker (Dryocopus pileatus)
Source: Front Parasitol. 2025 Mar 21;4:1547153. doi: 10.3389/fpara.2025.1547153 (PMC11968718; doi:10.3389/fpara.2025.1547153)

**Supplemental Figure 1.** Phylogenetic tree produced by FastTree v2.1 for partial cytochrome c oxidase subunit 1 gene sequences of a *Diplotriaena* sp. from a northern flicker (*Colaptes auratus*) and related species. The northern flicker sample is in bold.


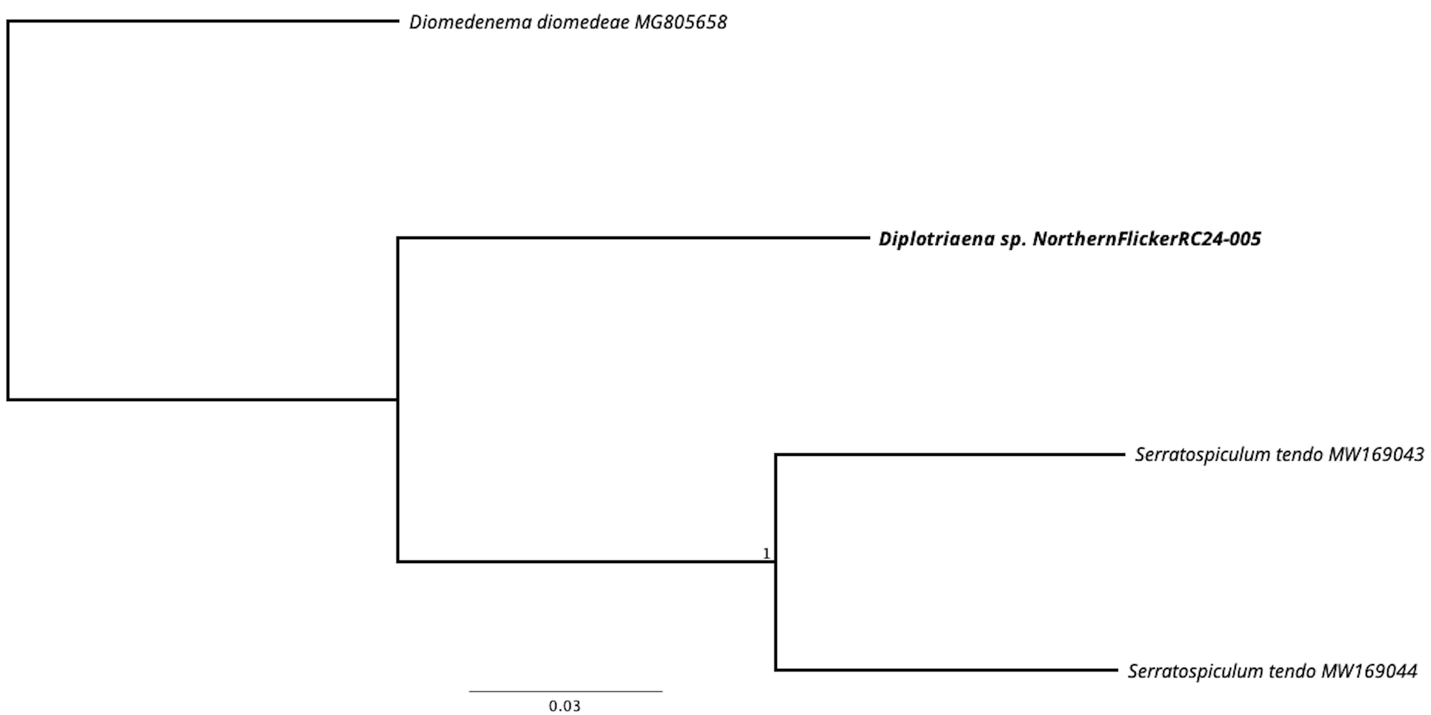

Supplement: Supplementary file 1 [file DataSheet1.docx]
