# Supplementary material for: Case Report: High burdens of air sac worms (Diplotriaena sp.) in three northern flickers (Colaptes auratus) and a pileated woodpecker (Dryocopus pileatus)
Source: Front Parasitol. 2025 Mar 21;4:1547153. doi: 10.3389/fpara.2025.1547153 (PMC11968718; doi:10.3389/fpara.2025.1547153)
Supplement: Supplementary file 2 [file Table1.docx]

**Table 1.** Measurements of morphological characteristics of the *Diplotriaena* specimens collected from a pileated woodpecker (*Dryocopus pileatus*) and a Northern flicker (*Colaptes auratus*) from Washington state, USA compared with similar and representative *Diplotriaena* species. Measurements in microns unless otherwise noted

|  | *Diplotriaena* sp. (n=32 females) | *Diplotriaena* sp. (n=23 females, 5 males) | *D. americana* | *D. serratospicula* | *D. obtusa* | *D. obtusa* | *D. tricuspis* | *D. anthreptis* | *D. bargusinica* | *D. utae* |
| --- | --- | --- | --- | --- | --- | --- | --- | --- | --- | --- |
|  | This study | This study | Walton, 1927 | Wehr, 1934 | Michalski et al., 2021 | Stanicka et al., 2021 | Anderson, 1959; Sonin, 1968; Morgan & Waller, 1940 | Dewi and Zhang, 2010 | Dutra Vieira et al., 2017 | Wong et al., 1983 |
| **Male** | Pileated woodpecker, *Dryocopus pileatus* | Northern flicker, *Colaptes auratus* | Northern flicker | Hispaniolan woodpecker, *Melanerpes striatus* | swallows, *Hirundo rustica & Petrochelidon pyrrhonota* | Eurasian blackcap, *Sylvia atricapilla* | corvids | brown throated sunbird, *Anthreptis malacensis* | chestnut-capped blackbird, *Chrysomus ruficapillus* | gray jay, *Perisoreus canadensis* |
| Length (mm) | ND* | 43-52 (46.4±3.6) | 40 | 33 | ND | 13-25 | 50-55 | ND | 21.96-35.05 | 16-18.8 |
| Trident length | ND | 75-125 (97.5±25.4) | 140-150 | 109.5 | 107-150 (129±11) | 123-152 | 90-120 | 100 | 100-140 (120±10) | 100-110 |
| Ant-nerve ring | ND | 190-210 (200±14.1) | ND | 146 | 173-427 (256±58) | ND | 200-300 | 140 | ND | 170-200 |
| total esophagus length (mm) | ND | 2.44-3.48 (2.96±0.74) | 3.5 | 2.77 | ND | ND | ND | ND | ND | 3.2-3.6 |
| anterior esophagus length (mm) | ND | 0.24-0.25 (0.245±0.007) | 0.25 | 0.22 | ND | ND | ND | ND | ND | 0.3 |
| posterior esophagus length (mm) | ND | 2.19-3.24 (2.72±0.74) | 3.25 | 2.55 | ND | ND | ND | ND | ND | 2.9-3.3 |
| Right spicule | ND | 620-1,000 (830±149.8) | 250 | 675 | 622-970 (719±85) | 484-660 | 800-1,100 | 560 | 410-520 (460±30) | 680-900 |
| Left spicule | ND | 1,070-1,400 (1,278±130.3) | 1,320 | 1,600 | 1,013-1,596 (1,288±121) | 630-838 | 1,140-2,000 | 910 | 680-760 (680±40) | 1,050-1,250 |
| **Female** |  |  |  |  |  |  |  |  |  |  |
| Length (mm) | 118-198 (163.4±22.5) | 140-210 (170.5±17.5) | 270 | 75 |  | 32-46 | 104-190 | 83-102 | 48.7-60.6 | 34.5-56 (42.4) |
| Trident length | 100-130 (118.5±12.2) | 67.5-92.5 (84±10.2) | 195-215 | 117 | 92-165 (127±15) | 128-157 | 90-120 | 120-130 (123) | 110-130 (115±6) | 115-140 (125) |
| Ant-nerve ring | 180-300 (221±47.2) | 180-210 (194±11.4) | 300 | 187 | 177-420 (240±51) | ND | 190-200 | 220-240 (220) | ND | 160-210 (193) |
| Ant-vulva | 400-600 (502±90.7) | 440-590 (535±71.4) | 1,020 | 300-375 | 585-1,075 (816±141) | ND | 430-760 | 560-750 (673) | 330-460 (390±50) | 400-620 (531) |
| total esophagus length (mm) | 3.11-4.15 (3.63±0.38) | 2.95-3.59 (3.18±0.24) | 3.81 | 4.874 | ND | ND | ND | ND | ND | 3.8-4.8 (4.3) |
| anterior esophagus length (mm) | 0.26-0.35 (0.31±0.038) | 0.23-0.33 (0.29±0.04) | 0.31 | 0.224 | ND | ND | ND | ND | ND | 0.2-0.3 (0.3) |
| posterior esophagus length (mm) | 2.82-3.8 (3.32±0.35) | 2.72-3.26 (2.89±0.21) | 3.5 | 4.65 | ND | ND | ND | ND | ND | 3.6-4.4 (4.0) |
| Egg length | 35-50 (48±4.9) | 40-50 (48±3.5) | 45 | 48-52 | ND | 46-48 | 45 | 36-42 | 40-52 (50±3.6) | 50-57 (54) |
| Egg width | 30-40 (35±4.2) | 35-40 (37.5±2.6) | 175** | 32-36 | ND | 28-30 | 35 | 29-31 | 25-37 (31±25) | 34-38 (37) |

* ND = No data; Ant-nerve ring = distance from anterior end to middle of nerve ring; Ant-vulva = distance from anterior end to vagina.

** eggs were reported to be "45 x 175 um" but it appears to be an error in the width measurement.

**References:**

Anderson, R.C. (1959). Preliminary revision of the genus *Diplotriaena* Henry and Ozoux, 1909 Diplotriaenidae: Diplotriaeninae. *Parassitologia.* 1, 195–307.

Dewi, K., Zhang, L. (2010) Two new species of spiruroid nematodes in birds from Kangean Island, Indonesia. *J Helminthol*. 84, 245-252.

Dutra Vieira, T., Pegoraro de Macedo, M.R., Fedatto Bernardon, F., Müller, G. (2017) Morphological, molecular and phylogenetic analyses of *Diplotriaena bargusinica* Skrjabin, 1917 (Nematoda: Diplotriaenidae). *Parasitol Int*. 66, 555-559.

Michalski, M. L., Kadolph, E., Roderick, C. L., Lankton, J. S., Cole, R. A. (2021). *Diplotriaena obtusa* (Nematoda: Diplotriaenidae) from barn swallows (*Hirundo rustica*) and cliff swallows (*Petrochelidon pyrrhonota*) collected during mortality events in the Upper Midwest, USA. *J Parasitol.* 107, 593-599.

Morgan, B.B., Waller, E.F. (1940). The occurrence of a rare filariid nematode from a crow. *Am. Midl Nat*. 24, 379-381

Sonin, M. D. 1968. Filariata of animals and man and the diseases caused by them. Part 2. Diplotriaenoidea. In Essentials of Nematology XXI, K. I. Skrjabin (ed.). Doklady Akademii Nauk Soviet Socialist Republics, Moscow, Union of Soviet Socialist Republics, 441 p. (in Russian, translated into English in 1975).

Stanicka, A., Zając, K. S., Jefimow, M., & Wojciechowski, M. S. (2021). *Diplotriaena obtusa* (Nematoda: Filariidae) infection in first-year *Sylvia atricapilla* from Poland – molecular evidence. *Eur. Zool. J*. *88*, 1144–1151. Doi:10.1080/24750263.2021.1998679

Walton, A. C. (1927). A revision of the nematodes of the Leidy collections. *Proc. Acad. Nat. Sci. Phila.* 79, 49-163.

Wehr, E. E. (1934). A new nematode of the genus *Diplotriaena* from a Hispaniolan woodpecker. *Smithsonian Miscellaneous Collections*. 91, 1-3.

Wong, P.L., Anderson, R.C., Frimeth, J. (1983) *Diplotriaena utae* sp. n. (Nematoda: Diplotriaenoidea) in the Gray Jay (*Perisoreus canadensis* (L.)) in Ontario, Canada. *Proc. Helminthol. Soc. Wash*. 50, 275-277
